# Flexural Behavior of Lightweight Sandwich Panels with Rice Husk Bio-Aggregate Concrete Core and Sisal Fiber-Reinforced Foamed Cementitious Faces

**DOI:** 10.3390/ma18081850

**Published:** 2025-04-17

**Authors:** Daniele Oliveira Justo dos Santos, Paulo Roberto Lopes Lima, Romildo Dias Toledo Filho

**Affiliations:** 1 Postgraduate Program in Civil Engineering, Federal University of Rio de Janeiro, Rio de Janeiro 21945-970, RJ, Brazil; daniele.justo@coc.ufrj.br; 2 Postgraduate Program in Civil and Environmental Engineering, State University of Feira de Santana, Feira de Santana 44036-900, BA, Brazil; prllima@uefs.br

**Keywords:** bending test, failure mode, stress–strain behavior, agricultural waste, alkaline treatment, sandwich panel

## Abstract

The development of sustainable and energy-efficient construction materials is crucial for mitigating the growing environmental impact of the building sector. This study introduces a new lightweight sandwich panel, featuring a core made of lightweight concrete with rice husk bio-aggregate (RHB) and faces constructed from foamed cementitious composites. The innovative design aims to promote sustainability by utilizing agro-industrial waste while maintaining satisfactory mechanical performance. Composites were produced with 4% short sisal fibers and matrices containing 15%, 20%, and 30% foaming agent. These composites were evaluated for density, direct compression, and four-point bending. It was found that the mixture with 20% foam volume demonstrated the highest efficiency for use in the production of sandwich panels. Concrete mixtures containing 50%, 60%, and 70% rice husk bio-aggregates were tested for density and compressive strength and used in the production of lightweight sandwich panels with densities ranging from 670 to 1000 kg/m^3^. Mechanical evaluation under flexion and shear indicated that the presence of fibers inhibited crack propagation in the face, enabling the creation of lightweight sandwich panels with deflection-hardening behavior. On the other hand, the increase in RHB content led to a reduction in the ultimate stress on the face, the core shear ultimate stress, and the toughness of the sandwich panels.

## 1. Introduction

Sandwich panels are modular construction systems that have been used in building construction in the form of external cladding, partitions, roofing and flooring, and fire-resisting walls. In recent years, the use of lightweight sandwich panels as building envelopes has increased to minimize energy consumption for artificial climate control. Buildings are responsible for 30–40% of energy consumption [1], with part of this energy being used to ensure thermal comfort in residential units, offices, and commercial buildings. Given the ongoing impacts of climate change, this trend is expected to continue in the coming years, which demands an urgent search for more sustainable building solutions.

Lightweight sandwich panels can serve as masonry elements in constructing new buildings or as retrofit systems for existing structures. These panels are designed to minimize temperature transfer between the external environment and the building’s interior. Typically, they consist of an insulating core material enclosed by strong, impermeable, cement-based faces. One of the most commonly used materials for the core of these panels is expanded polystyrene (EPS) [2]. Despite its light weight, with a density of approximately 0.03 g/cm^3^, and low thermal conductivity, EPS has notable environmental drawbacks due to the materials and processes used in its manufacture. Its production relies on non-renewable materials, involves high energy consumption, and generates greenhouse gas emissions. Furthermore, disposing of EPS at the end of a building’s lifecycle presents challenges due to its non-biodegradability. Therefore, various other materials have been studied as insulation elements for the core of lightweight sandwich panels in buildings, such as coconut fiber [3], phase change material [4], fiber-reinforced foamed concrete [5], or lightweight concrete containing bio-aggregate [6].

The incorporation of bio-aggregates into concrete presents several advantages by using renewable source materials as substitutes for mineral-origin aggregates. Additionally, bio-aggregates present lower energy costs and generate less waste during production. From a mechanical perspective, studies demonstrate that concretes with bio-aggregates exhibit sufficient flexural and compressive strength to be used as insulation elements [7,8,9].

Moussa et al. [10] investigated the mechanical, thermal, and acoustic properties of a coffee husk-based composite. Their findings indicated that the material exhibited sufficient mechanical performance for non-load-bearing structures, making it a suitable option for manufacturing construction bricks with enhanced acoustic insulation. Bio-aggregates can be produced from agro-industrial waste, such as wheat husk and hemp hurd [11] and rice husk waste [12], thereby increasing the circular economy. The use of rice agro-industrial waste is strategic worldwide, given that its production is millions of tons per year. For each ton of rice produced, 24% is the husk, which has no commercial value and is typically used as an alternative heat source for generating hot gases to dry the rice in processing plants [12]. Due to its high silica content, the ash resulting from the burning of rice husk has been successfully used as a mineral addition to concrete [13].

Rice husk waste has also been utilized as a substitute for mineral aggregates in concrete, producing a material with adequate mechanical strength and reduced density [14]. This makes it possible to create lightweight concrete with densities lower than those of conventional concrete [15], which makes it suitable for use as the core material in sandwich panels. In addition to enhancing thermal and acoustic comfort, incorporating rice husk as a bio-aggregate in concrete walls helps to reduce GHG emissions compared with walls made with conventional concrete or ceramic blocks [16]. However, achieving the desired performance requires a precise mix design, as the physical and mechanical properties of lightweight concrete are highly dependent on the bio-aggregate content.

The faces of lightweight sandwich panels made from cement-based materials are typically reinforced with stiffening elements to ensure sufficient mechanical resistance during transportation, assembly, and building use. Steel mesh reinforcement can be used, but it has limitations regarding the corrosion of the reinforcements [17], which requires increasing the cover layer and, consequently, the panel thickness. As an alternative, fiberglass mesh [18], aramid fabric [19], or basalt textile fiber mesh [20] can provide sufficient resistance for the faces.

Pennarasi et al. [21] investigated the incorporation of coconut fibers into concrete mixtures at various ages and dosages. Their findings revealed a notable enhancement in mechanical properties, particularly in applications such as concrete pavers. Similarly, Quintero Garcia [22] studied the use of coconut coir fiber to strengthen concrete, emphasizing its potential to improve both durability and overall structural performance. More sustainable solutions have been presented with the use of vegetable fibers and fabrics as reinforcement for cementitious composites that have been used on the faces of sandwich panels [7,23,24].

Vegetable fiber-reinforced composites for use as construction elements have been extensively studied in recent years since the mechanisms of fiber degradation in an alkaline environment were identified and appropriate solutions were implemented [25]. A challenge in using these composites as face elements of sandwich panels is the higher density of the cementitious matrix, which means that the panel, even with a lightweight core, does not have the required lightness and limits its application. In this regard, the development of fiber-reinforced foamed concrete (FRFC), with densities ranging from 300 to 1850 kg/m^3^ [26], shows great potential for application in lightweight sandwich elements. Produced with manufactured fibers or natural fibers [5,26], the properties of FRFC, on the other hand, depend on the type and content of the fiber used, as well as the amount of foam added.

Studies [27] emphasize the growing interest in sustainable construction practices and the potential of biomaterial-infused concrete in engineering. These studies contribute to expanding knowledge and advancing the use of biomaterials in concrete technology. Furthermore, they highlight the importance of plant residues as sustainable construction materials, offering effective alternatives to synthetic and mineral-based reinforcements while promoting waste reutilization and environmental sustainability.

The objective of this study is to evaluate the mechanical behavior of lightweight sandwich panels with a core made of concrete incorporating rice husk bio-aggregate (RHB) and cementitious foamed faces reinforced with vegetable fibers. This research specifically evaluates key performance parameters, including flexural strength, shear resistance, failure mode, and toughness through four-point bending tests. To achieve this, concrete mixtures incorporating different percentages of rice husk residue and foamed composites with varying foaming agent amounts were developed and tested. By analyzing the effects of RHB content and foaming agent variations, this study aims to identify the optimal composition for improved structural integrity and sustainability in construction applications.

## 2. Materials and Methods

### 2.1. Sisal Fiber-Reinforced Foamed Cement Composite (SFRFCC)

**Figure 1:**
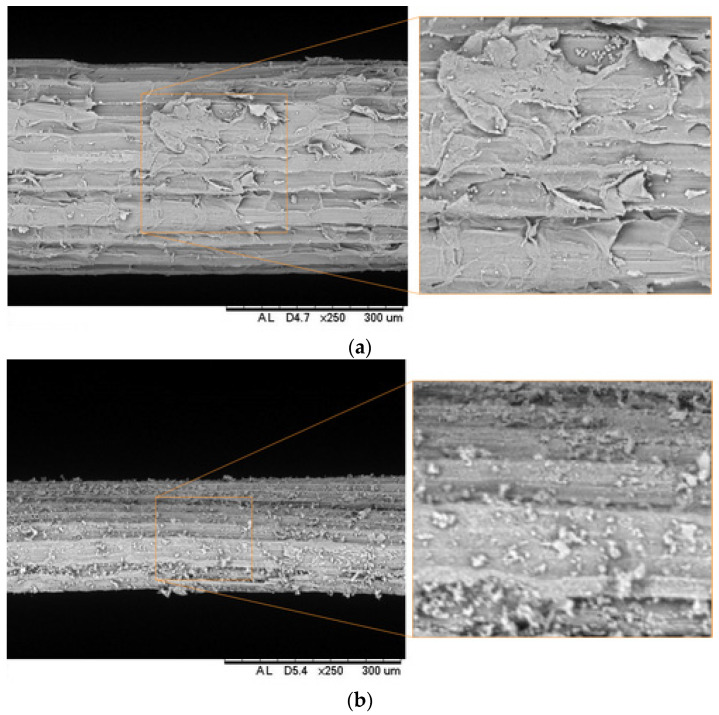
Effect of treatment on fiber surface: (**a**) natural fiber; (**b**) treated fiber.

Sisal (*Agave sisalana*) fiber, sourced from Valente City, Brazil, was used in this research. According to [28], the plant has smooth leaves approximately 10 cm wide and 150 cm long. Sisal fiber has demonstrated excellent mechanical performance, with a tensile strength of 484 MPa and a Young’s modulus of 19 GPa [29]. Initially, the sisal fibers were washed in hot water (T~50 °C) for 1 h and then washed in running water. After this, the fibers were aligned using a “nail comb”, cut to a length of 40 mm using scissors, and then subjected to an alkaline treatment. Approaches such as alkali treatment have been proposed for their potential to improve the tensile strength of natural lignocellulosic fibers [30]. This treatment consisted of immersing the fibers for 50 min in a 0.73% solution of calcium hydroxide Ca(OH)_2_ produced in the laboratory with deionized water. According to [31], immersion periods of 30 to 60 min in low alkali concentrations (0.5–1%) do not degrade natural fibers. This method was successfully employed in [32]. Alkaline treatment promotes the superficial cleaning of the fiber, and it is possible to observe that the fibrils become more individualized and more evident. In Figure 1, natural fiber and treated fiber surfaces are compared; it is possible to observe a more irregular surface of the fiber, suggesting that calcium hydroxide deposition occurred. These similarities were also found by [32].

The foamed cement matrix, with a mix design of 1:1:0.50 (binder: sand: water/binder ratio), was produced with three different contents of foam agent (Eco-foam-Air): 15%, 20%, and 30% foam, calculated based on the volume of the produced matrix. It is a synthetic-based, chloride-free, and biodegradable liquid additive that generates high-density, durable, and long-lasting foam while maintaining a structure of micro- and nano-air bubbles. This enables the production of cellular concrete, lightweight concrete, and lightweight mortar with densities ranging from 400 kg/m^3^ to 1900 kg/m^3^ by preventing communication between air bubbles. The binder composition consisted of 50% high early strength ordinary Portland cement, 10% fly ash, 30% metakaolin, and 10% silica fume. This proportion of fly ash, metakaolin, and silica fume ensures the durability of the fibers, as it forms a matrix free of calcium hydroxide. The superplasticizer and viscosity-modifying agent were added at dosages of 1% and 0.1%, respectively, calculated based on the mass of the binders.

The mixture was produced in a laboratory under controlled temperature conditions (21 °C ± 2 °C) using a mixer with a capacity of 20 dm^3^. The mixing procedure was carried out as follows: the binders were homogenized in the mixer for 1 min and 30 s, followed by the continuous addition of water (pre-mixed with the superplasticizer) over 3 min. The mixing process was then paused for 30 s to scrape off any material adhering to the mixer walls. After this, sand was gradually added over 2 min. Finally, fibers were incorporated into the mixture, producing composites with a fiber volumetric fraction of 4%.

For the foamed composites, the foam was separately prepared using a 20 dm^3^ mixer with 200 mL of water and a foaming agent. It was then incorporated into the composite mixture for approximately one minute, with the uniformity of foam distribution visually assessed based on the aerated appearance of the mixture. The resulting matrix in this study exhibited a spread value of 400 mm.

The SFRFCC specimens were evaluated for their physical and mechanical properties. Compression strength tests and four-point bending test were conducted on a Shimadzu AGX-100 kN electromechanical testing machine (Kyoto, Japan) with displacement control set at 0.3 mm/min. For the uniaxial compression strength test, axial displacements were measured using two linear variable differential transformers (LVDTs) positioned in the central zone of the sample. In the four-point bending test, the load application points were spaced 100 mm apart, with a span length of 300 mm. Data acquisition was performed using Trapezium software (version 1.4.0), which also facilitated the measurement of mid-span deflection via an integrated LVDT. All tests were performed under the following conditions: 50 ± 5% relative humidity and a temperature of 23 ± 2 °C.

### 2.2. Rice Husk Bio-Agreggate Concrete (RHBC)

**Figure 2:**
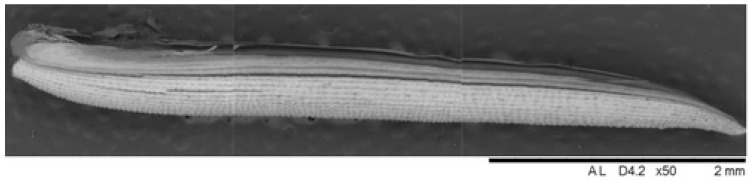
Geometric shape and surface detail of rice husk.

In this study, lightweight concrete was produced using rice (*Oryza sativa*) husk as a bio-aggregate, replacing traditional mineral aggregates. The rice husk used as a bio-aggregate was sourced from local farmers in the municipality of Mogi das Cruzes, Brazil. It had a specific mass of 0.3 g/cm^3^, classifying it as a lightweight aggregate, and a moisture content of 12%. The water absorption capacity of the rice husk was approximately 114%. The RHB had an elongated shape and a rough surface texture, as shown in Figure 2, which provide a good mechanical bond when used as a bio-aggregate in concrete, but also enhance its water absorption properties.

Rice husk bio-aggregate concrete (RHBC) was produced with 50%, 60%, and 70%, in volume, RHB content. The binder consisted of 60% high early strength ordinary Portland cement, 10% fly ash, and 30% metakaolin. The viscosity-modifying agent Rheomac UW 410 (VMA) and potable water were used. Calcium chloride CaCl_2_ was incorporated as the setting accelerator. To compensate for the water absorption of the bio-aggregate, additional water content was added to the mixture. The mix proportions for the RHBC are presented in Table 1.

The density of the concrete was evaluated based on the relationship between the mass and volume of cylindrical specimens (with a diameter of 100 mm and a height of 200 mm) in their dry state. The mechanical properties were assessed through uniaxial compression strength tests on these cylindrical specimens using a methodology similar to that applied for composite materials.

To produce the rice husk bio aggregate concrete, a 20 dm^3^ mixer was used. Initially, the RHB was pre-mixed with binding materials in the same bag used for weighing the materials. After this, the mixture was transferred into the mixer. With the mixer running, the total amount of water was added slowly over 1 min and 30 s, with a 30 s pause to remove material adhered to the sides of the mixer bowl. After 5 min of mixing, the VMA was added. The total mixing time was 8 min.

### 2.3. Lightweight Sandwich Panels (LSP)

**Figure 3:**
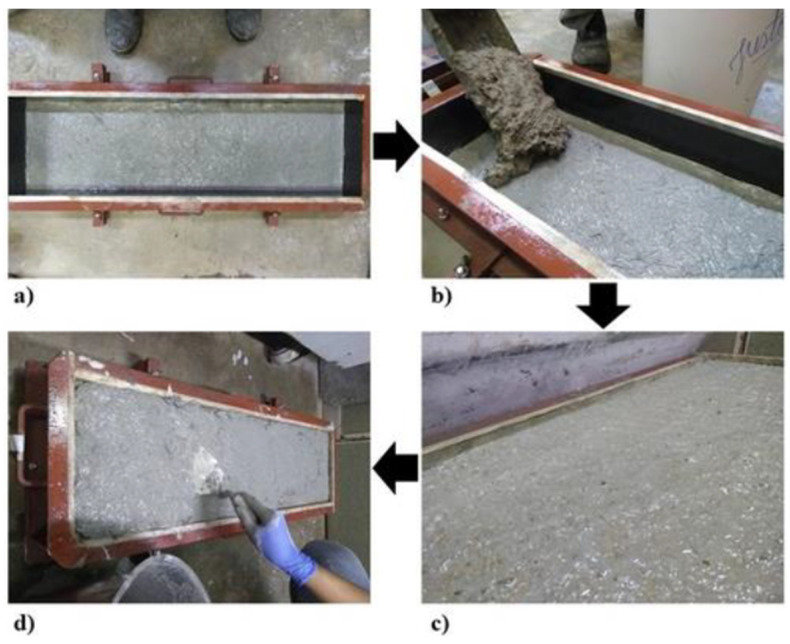
Production process of sandwich panel: (**a**) Placement of the bottom face; (**b**) Placement of the core; (**c**) Detail showing the thickness occupied by the core; (**d**) Placement of the top face 8 h after molding the core.

The sandwich panels were produced using a steel mold with dimensions of 900 mm × 25 mm. The production process, shown in Figure 3, involved the following steps: molding the bottom face with a thickness of 1 cm, molding the rice husk concrete core with a thickness of 8 cm, and molding the top face with a thickness of 1 cm.

The panels were demolded after 24 h and cured at 21 ± 3 °C with a relative humidity of 65% for 28 days prior to testing. Two configurations of four-point bending tests were performed to evaluate the mechanical behavior of the lightweight sandwich panel under flexion or shear.

**Figure 4:**
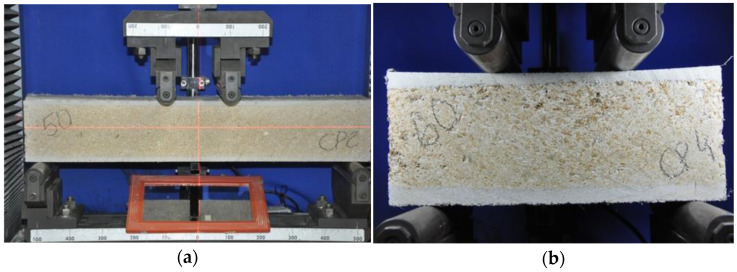
Setup of (**a**) bending test; (**b**) shear test.

The first test, conducted according to ASTM D7249, aimed to determine the facing properties of sandwich panels subjected to flexure. Samples with dimensions of 550 mm × 80 mm × 100 mm (length × width × height), a support span length of 500 mm, and a loading span length of 100 mm were used (Figure 4a). Once the force–displacement diagram is obtained, the ultimate stress on the face (F_u_) is given by Equation (1)

F_u_ = [P_max_·(S − L)]/[2·(d + c)·b·t] (1)

where P_max_ = maximum force prior to failure, S = support span length, L = loading span length, d = measured sandwich total thickness, c = calculated core thickness, b = specimen width, and t = nominal facing thickness.

The second test, conducted according to ASTM C393, aimed to determine the core shear properties of the lightweight sandwich panel subjected to flexure. Samples with dimensions of 250 mm × 50 mm × 100 mm (length × width × height), a support span of 200 mm, and a load span of 100 mm were used (Figure 4b). This configuration corresponds to a quarter-point load setup, with the distance between the support and the load point equal to one-quarter of the free span. From the test results, it is possible to calculate the core shear ultimate stress (F_s_) using Equation (2):

F_s_ = P_max_/[(d + c)·b] (2)

where P_max_ = maximum force prior to failure, d = measured sandwich total thickness, c = calculated core thickness, and b = specimen width.

These samples were tested on a Shimadzu 100 kN universal testing machine at a displacement control speed of 0.3 mm/min, with load application points spaced 100 mm apart. The central deflection was measured using an LVDT connected to the device.

## 3. Results

### 3.1. Characterization of Sisal Fiber Reinforced Foamed Cement Composite

**Figure 5:**
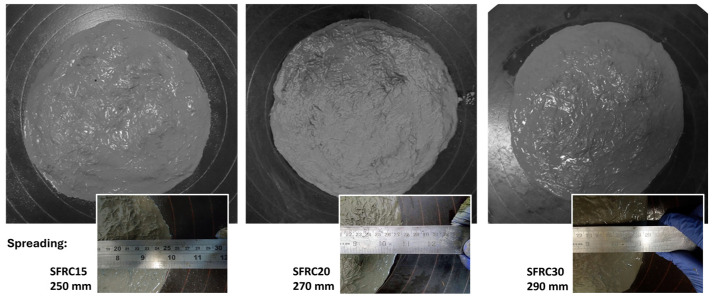
Visual appearance and workability measurement of the composites.

Figure 5 illustrates the appearance and workability of foamed mixtures subjected to the flow table test, in accordance with ASTM C1437. Visual analysis of the mixtures revealed the presence of uniformly distributed air bubbles within the matrix, with a higher concentration observed in the SFRC30 sample, which contained 30% foam agent.

The workability of the mixtures, evaluated based on the spread shown in Figure 5, ranged from 250 mm to 290 mm, increasing as the foam content increased from 15% to 30%. An increase in cement mortar workability due to the inclusion of 20% to 35% foam agent was also observed by Othman [33].

This improved workability induced by the foam agent contributed to the homogenization of fiber-reinforced mortar. The presence of bubbles facilitated fiber dispersion, preventing agglomeration and promoting a more uniform distribution. As the volume of added foam increased, the mixture exhibited greater diameter flow, indicating enhanced fluidity of the composite.

**Figure 6:**
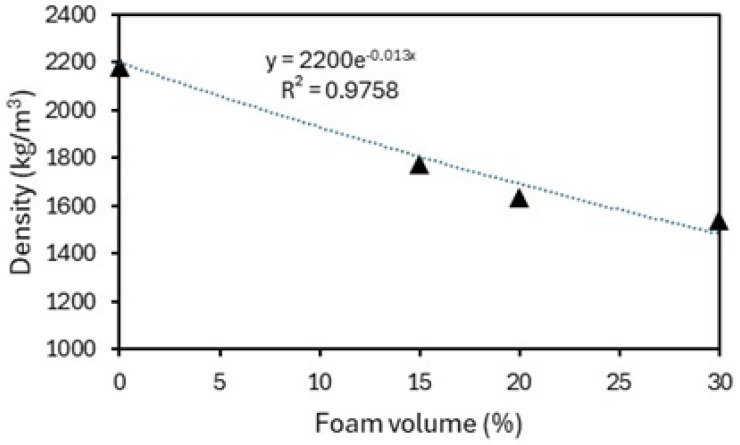
Effect of foam volume on density of composites.

Figure 6 illustrates the effect of the addition of a foaming agent on the density of the composites reinforced with sisal fibers; from the experimental results (black triangles), an exponential relationship is observed, with a density reduction of up to 29.6% for a foam content of 30%. Foamed composites reinforced with polypropylene fibers [34] showed a similar relationship between density and foam content, which was associated with the increased air voids created by the addition of the foaming agent. These voids were observed by the authors through scanning electron microscopy.

**Figure 7:**
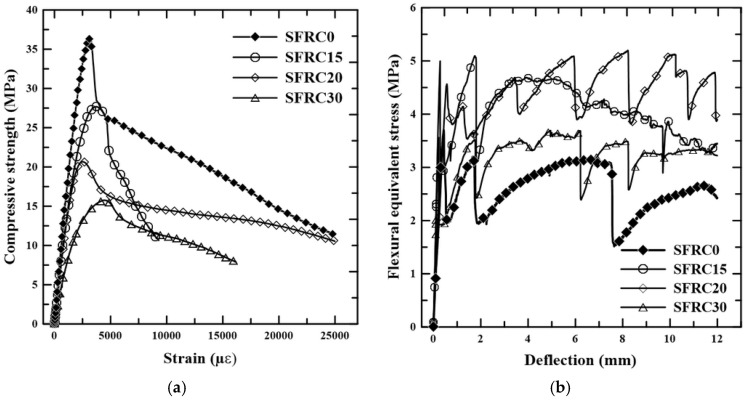
Mechanical behavior of composites: (**a**) compression stress–strain curves; (**b**) bending stress–deflection curves.

The incorporation of voids into cement-based materials has an immediate effect on their mechanical properties. The strength of concrete is closely linked to its porosity; greater porosity creates more voids in the cement matrix, weakening the material’s load-bearing capacity. Additionally, porosity impacts the elastic modulus, as an increase in pores disrupts the continuity of the solid phase, reducing the concrete’s resistance to deformation under stress. Figure 7a shows the typical stress–strain curves of the composites under uniaxial compression, while Figure 7b displays the stress–deflection curves of the composites under bending. Table 2 presents the mean values and the coefficient of variation (in %) of the mechanical properties obtained from five samples.

Observing the results, it can be seen that there was a reduction in the compressive strength and modulus of elasticity of the lightweight composites as the percentage of foam increased. The reductions in compressive strength, relative to those of the conventional composite SFRC0, were 17.4%, 37.9%, and 54.1% for SFRC15, SFRC20, and SFRC30 mixes, respectively. The effect of foam content on the modulus of elasticity was similar, with a reduction of up to 50.6% for a foam content of 30%. According to [34], the reduction in the compressive strength of lightweight composites is associated with the pore content created by the addition of the foaming agent.

For the composites under bending, on the other hand, an increase in flexural strength is observed with the incorporation of foam volume. Figure 7b shows that, after the first crack appeared in the composite, the fibers redistributed internal stresses, leading to an increase in the material’s load-bearing capacity as new cracks formed. This process is known as multiple cracking.

**Figure 8:**
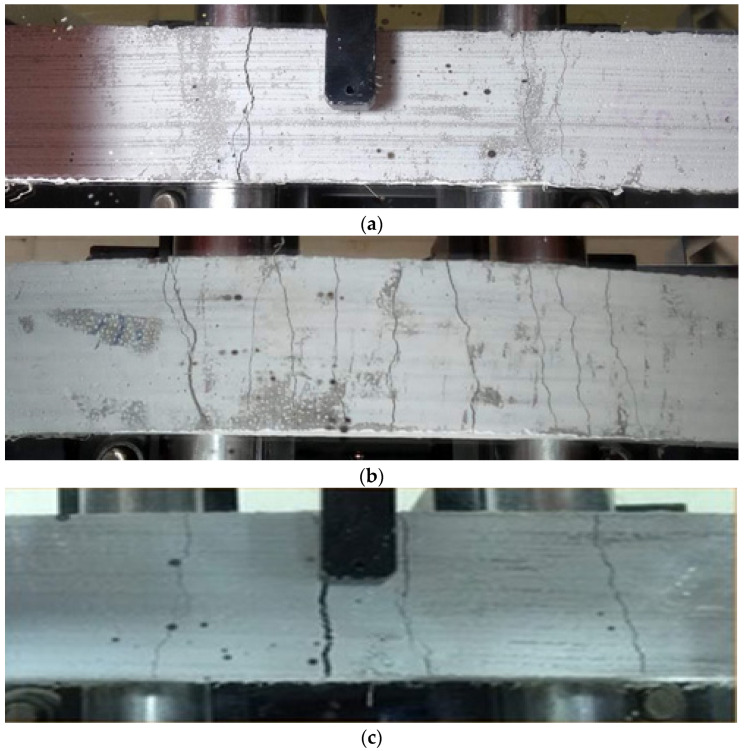
Cracking pattern of composites: (**a**) SFRC15, (**b**) SFRC20, and (**c**) SFRC30.

Figure 8 shows the crack pattern of the composites, where it can be observed that the foam agent content in the mixture influenced the formation and distribution of cracks. At the end of the test, the formation of 3, 11, and 4 cracks was observed in the composites for foam agent contents of 15%, 20%, and 30%, respectively. The formation of more cracks was linked to the presence of entrained air, which facilitated the appearance and development of cracks. The introduction of the foaming agent also enhanced the workability of the mixtures and promoted better fiber homogenization. A similar behavior was noted by [35] in foamed concrete with basalt fibers. However, in the case of the SFRC30 mixture, the excessive air voids limit the stress transfer between the fibers and the matrix, as observed by [36] in their studies.

Multiple cracking led to an up to 33% increase in the post-cracking flexural strength for a foam volume of 20% compared with the conventional composite. Similar increases in tensile and flexural strength were observed in other studies involving foamed composites with both natural and manufactured fibers [37].

**Figure 9:**
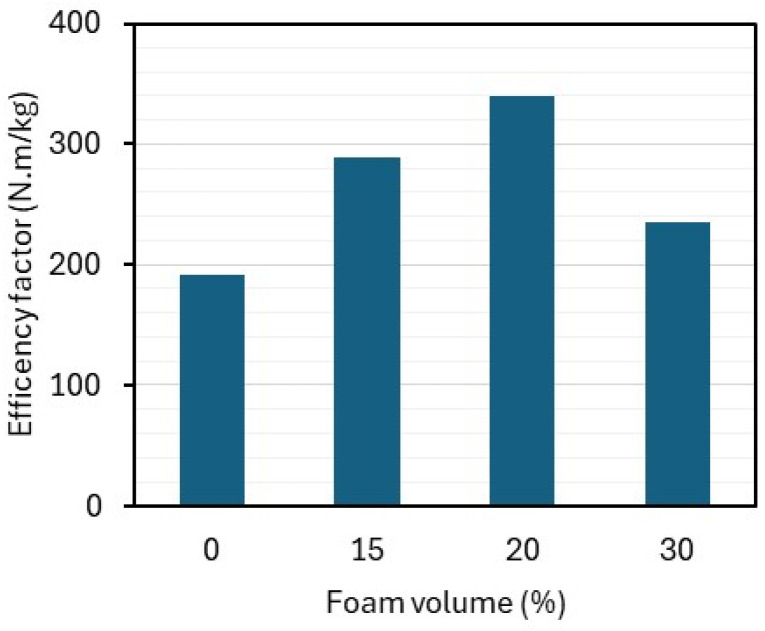
Efficiency factor of composites under flexion.

Figure 9 shows the efficiency factor of the composites, defined as the ratio of flexural strength to the density of each mixture. This concept, applied by Moravia [38] for the evaluation of lightweight concrete, indicates that a composite with 20% foam volume demonstrates the highest efficiency for use in the production of sandwich panels.

The bending behavior of the composites supports the use of the mixture SFRC20 on the faces of the sandwich panel, as these elements are going to be responsible for resisting compressive and tensile stresses under bending and to prevent sudden panel failure.

### 3.2. Characterization of Rice Husk Bio-Agreggate Concrete (RHBC)

**Figure 10:**
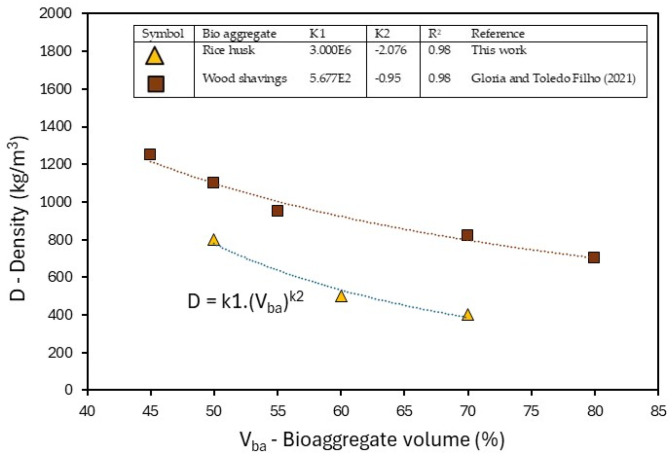
Effect of type and volume of RHB on density of concrete: comparison between experimental results and literature data [7].

Figure 10 shows the effect of the RHB content on the density of the concrete. The reduction in density occurs due to the introduction of the highly porous RHB into the cementitious matrix. The replacement of natural aggregate with different bio-aggregates confirms this hypothesis, showing a reduction in density with the crescent amount of bio-aggregate used [16,39].

The density of bio-aggregate concrete is typically influenced by the type and content of bio-aggregate. An exponential relationship with the density, which can be expressed as follows, was proposed by [7] for concrete with wood shaving bio-aggregates:

D = k1 (V_ba_)^k2^ (3)

where D is the density of concrete with bio-aggregate, V_ba_ is the bio-aggregate volume, and k1 and k2 are the constant coefficients to be adjusted depending on the bio-aggregate type and mixture proportion parameters.

In Figure 9, the coefficients k1 and k2 obtained for the RHBC are presented in comparison with a bio-aggregate concrete produced with wood shavings [7]. It shows the same exponential relationship between the density and the volume of the concrete, but with an expressive reduction in density for the same content of rice husk.

Another relevant factor to consider regarding the effect of RHB on density reduction is its influence on the workability of concrete. The addition of RHB alters workability, resulting in increased air incorporation during the production process, similar to what is observed with other types of lightweight aggregates [40]. In this study, the increase in RHB content reduced the spread of the fresh mix from 240 mm (50% bio-aggregate) to 190 mm (70% bio-aggregate). The mixtures presented, however, adequate workability and good mouldability.

**Figure 11:**
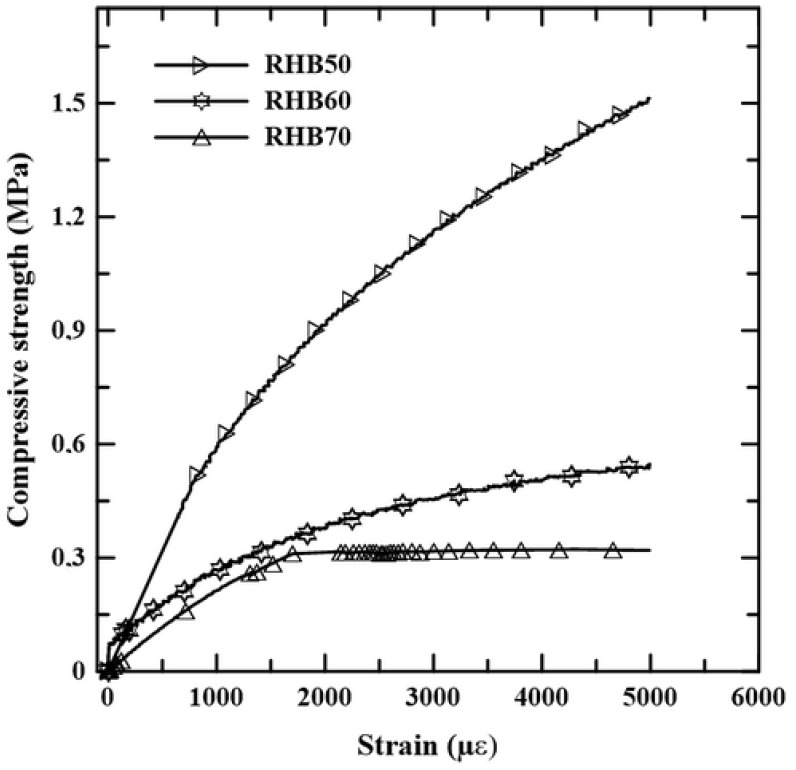
Stress–strain curves of RHBC under compression.

Figure 11 presents the stress–strain curves of RHBC under axial compression. The average compressive strength of RHBC was 1.92 MPa, 0.50 MPa, and 0.35 MPa for concretes containing 50%, 60%, and 70% rice husk bio-aggregate, respectively. Similar findings from other researchers [41] indicate that lightweight core materials generally exhibit low mechanical strength under compression loads. However, the low compressive strength does not restrict the use of RHBC in the production of sandwich panels for thermal and acoustic insulation. According to the PCI Committee Report [42], various materials can be used as cores for precast concrete sandwich panels, with compressive strengths ranging from 5 to 100 psi (0.03 to 0.70 MPa). This is because the mechanical strength of sandwich panels is primarily provided by their rigid face layers.

The elastic module of the RHBC also decreased with the increase in rice husk content, but a very high strain at failure was observed for all mixtures.

### 3.3. Mechanical Performance and Failure Mode of Lightweight Sandwich Panel (LSP)

Sandwich panels with faces made of sisal fiber-reinforced foamed composites were produced with three types of cores made from RHBC containing 50%, 60%, and 70% RHB. The density values of the sandwich panels, evaluated by dividing the dry mass by the total volume, were 1000 kg/m^3^, 840 kg/m^3^, and 670 kg/m^3^, respectively. These values indicate that all the panels can be classified as lightweight sandwich panels, with the potential to provide thermal comfort in buildings. The very low density of the mix containing 70% RHB is notable.

The mechanical performance of LSP was evaluated through four-point bending tests conducted under two configurations: one to characterize the facing properties (ASTM D7249) and the other to assess the core shear properties (ASTM C393).

#### 3.3.1. Evaluation of the Facing Properties of LSP by Long Beam Flexure

**Figure 12:**
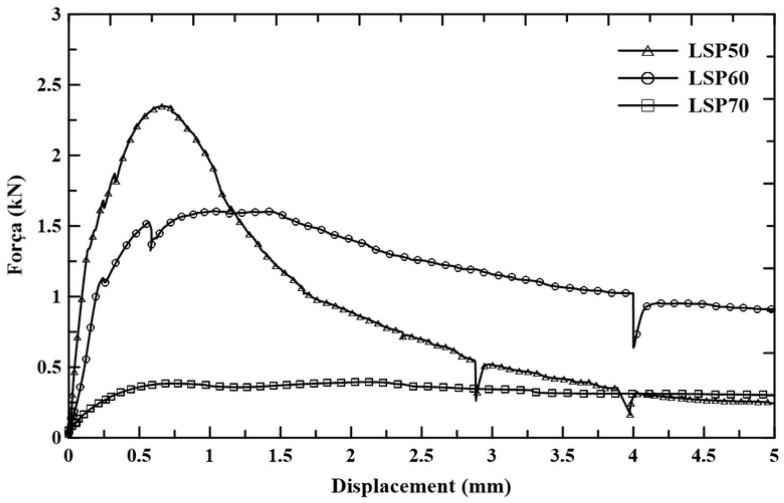
Typical force–displacement curves of lightweight sandwich panels under bending.

Figure 12 shows the typical force–displacement curves of sandwich panels tested to failure under normal tensile stresses according to ASTM D7249 with a span length of 500 mm.

The mechanical behavior of sandwich panels is initially linear until the first crack appears on the lower face. The fibers reinforcing the composite create a stitching effect on the initial crack, preventing its further propagation. This results in a higher resistance force in the panel, demonstrating deflection-hardening behavior until it reaches its peak resistance. As noted by Dey [43], this behavior describes a material’s ability to withstand increasing loads and deformation after the first crack owing to the fiber-bridging effect. This enhances the load-bearing capacity beyond the initial crack, improving both toughness and ductility.

After this peak, the behavior shifts to deflection–softening, with a gradual decline in resistance capacity. Similar behavior was noted by Frazão [23] for sandwich panels with cement faces reinforced with short sisal fibers.

**Figure 13:**
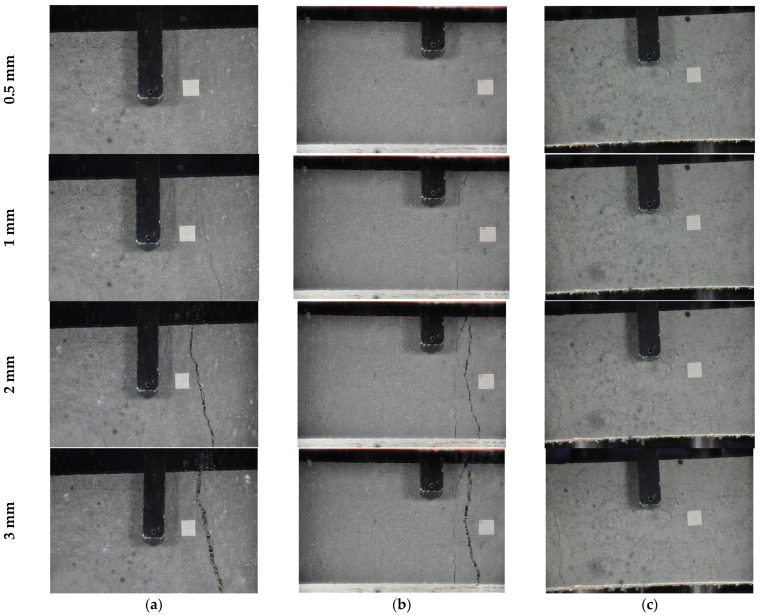
Cracking pattern of bottom face of lightweight sandwich panels: (**a**) LSP50, (**b**) LSP60, (**c**) LSP70.

Figure 13 illustrates the cracking pattern on the bottom face of the LSP at various displacement stages: 0.5, 1.0, 2.0, and 3.0 mm. Up to a displacement of 0.5 mm, no visible cracks are observed on the LSP face. However, the deviation from linearity in the force–displacement curves suggests the appearance of micro-cracks at displacements between 0.1 and 0.25 mm, indicating the initiation of cracks. At a displacement of 1 mm, two cracks are visible on the faces of the LSP50 and LSP60 laminates, while one crack is seen in the LSP70 laminate. After the peak, as displacement continues to increase, only crack openings are observed in all lightweight sandwich panels until failure occurs. This process is marked by the pull-out of fibers from the matrix and the propagation of the crack through the core of the panel.

Table 3 presents the first crack force on the face (F_fcr_), the maximum force (F_max_) in the lightweight sandwich panel, the ultimate stress on the face (F_u_), and the toughness, which is calculated as the area under the force–displacement curve for a displacement of 5 mm.

The first crack force values ranged from 0.28 kN to 1.08 kN. Although it was related to the tensile strength of the composite faces, the variation in values indicates that the lower stiffness of the LSP70 core led to greater internal deformations in the faces, which reached the cracking strain limit. Indeed, in sandwich panels with shear connector inclusion, an increase in flexural strength was observed, which suggests a direct relationship between the stiffening of the core and the development of stresses in the faces [44].

After the first crack, the panels exhibited a deflection–hardening behavior, with increases in the load-carrying capacity of 2.21 kN, 1.62 kN, and 0.31 kN for the LSP50, LSP60, and LSP70 panels, respectively. The maximum loads occurred at small deflection values of up to 1 mm, which corresponded to a deflection of L/500 (L = span length). After the peak, the panels showed a deflection–softening behavior, like that observed in composites with faces reinforced with short sisal fibers and a core made of bioconcrete [23].

The maximum load-bearing capacity is also directly affected by the strength of the RHBC core. The LSP70 panel, with a core having lower compressive strength, showed an 85% reduction in maximum force and 86% reduction in flexural strength (F_u_) compared with the LSP50 panel. Gloria [7] also noted a threefold reduction in maximum flexural load when increasing the bio-aggregate content of wood shavings in the sandwich panel, indicating that the stronger core was more effective in increasing the flexural strength of the sandwich system.

The LSP50 panel exhibited higher toughness values, associated with greater resistance to crack propagation through the core of the high-strength RHBC. Increasing the volume fraction of RHB reduces the amount of matrix that is responsible for governing the strength of concrete. Factors of greater influence, such as a high total water/cementitious materials ratio, a smaller amount of paste in RHBC with a high volumetric fraction and RHB morphology tend to produce concretes with a lower capacity to resist mechanical loads.

An important contribution of the fibrous reinforcement on the face of the sandwich panel is maintaining mechanical strength after the peak load, preventing brittle failure of the panel, and increasing its toughness. Table 3 indicates that toughness is inversely proportional to the RHB content in the core; the toughness of LSP60 and LSP70 panels were 12% and 76% smaller, respectively, than the toughness of the panel LSP50. The effect of the core material on the energy-absorbing capacity of the sandwich panel was also highlighted by [20], who observed that a polymeric core (EPB) exhibited greater energy dissipation capacity than a conventional concrete core.

#### 3.3.2. Evaluation of the Core Shear Properties of LSP by Beam Flexure

**Figure 14:**
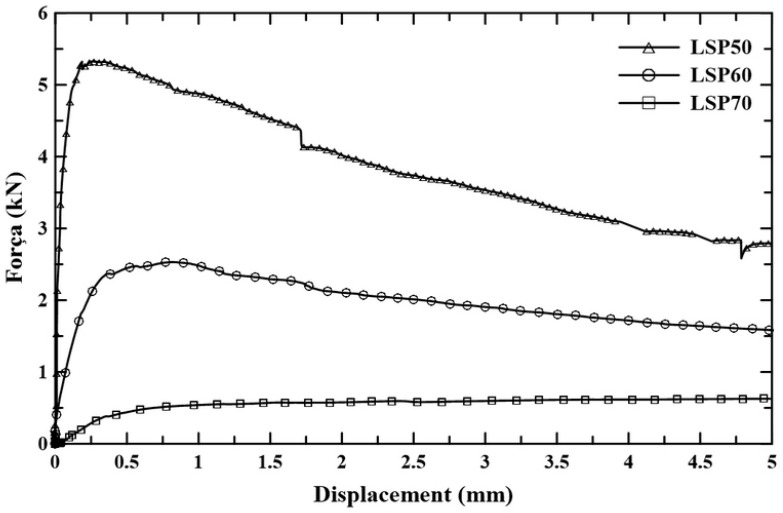
Typical force–displacement curves of lightweight sandwich panels under shear.

Figure 14 shows the typical force–displacement curves of sandwich panels tested to failure under shear stress according to ASTM C393.

**Figure 15:**
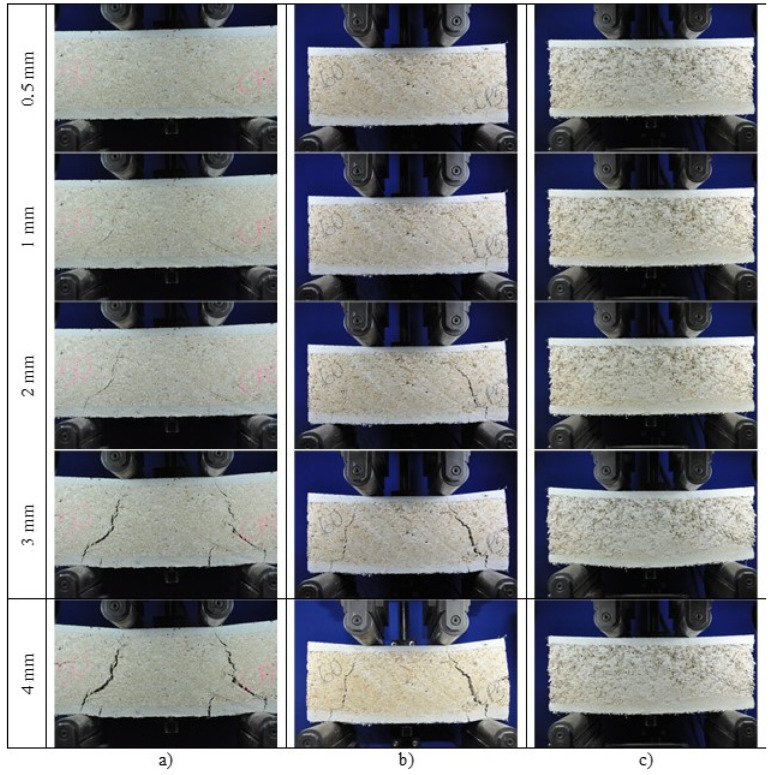
Cracking propagation of lightweight sandwich panel under shear with: (**a**) 50%, (**b**) 60%, and (**c**) 70% bio-aggregate.

The force–displacement curves of the lightweight sandwich panels under shear (Figure 14) showed an increase in load resistance until reaching a peak maximum value, followed by a gradual decrease in resistance. This reduction in load is caused by the formation of a shear crack in the core of the panel, as shown in Figure 15.

At a displacement of 0.5 mm, small cracks can be observed on the right support of LSP50 and LSP60, but not on the sandwich LSP70. As displacement increases, the initial cracks widen, and new cracks form on the left support. Cracks in the face at the support device are controlled by the presence of sisal fiber reinforcement. At a displacement of 1 mm, the shear cracks extended through the core, and cracks appeared in the bottom faces below the load application point. Despite the progression of cracking in both the core and the face, the panels maintained their load-bearing capacity due to the stress transfer in the face cracks facilitated by the sisal fiber composite. As a result, the panel failure was ductile and occurred after large displacements. However, the toughness values decreased as the RHB content in the core concrete increased.

The main difference between sandwich panels and monolithic panels lies in the significant shear deformation [45], which makes the core play a crucial role in the mechanical behavior. When the core has higher stiffness and stronger core-to-face adhesion, internal forces are transferred between the faces, allowing the sandwich panel to function as a fully composite structure [46]. The core of the LSP50 laminate exhibits greater stiffness and resistance to shear loads, stabilizing the faces against wrinkling or buckling and ensuring that the facings remain properly separated without sliding relative to each other [47]. However, when the core stiffness is reduced, as seen in the LSP60 and LSP70 laminates, a lack of necessary shear strength is observed. This results in the two thin facings acting as independent panels and losing the sandwich effect [46]. These sandwich panels are then considered to have a non-composite mechanical behavior because the core cannot effectively transfer longitudinal shear. This behavior has led to the increased use of shear connectors in sandwich panels with lightweight cores [48].

Table 4 presents the maximum force (F_max_), the core shear ultimate stress (Fs), and the toughness of lightweight sandwich panels.

The influence of the RHB content of the core on mechanical strength Fs is like the observed ultimate stress on the face F_u_ (Table 3), with an 87% reduction in maximum load resistance for LSP70 compared with LSP50. This is because the core strength is reduced with the RHB content, with core shear ultimate stress (F_s_) values ranging from 0.37 MPa to 0.05 MPa. The change in core strength directly affects the toughness of the sandwich panels, resulting in reductions of 40% and 83% for the LSP60 and LSP70 panels, respectively, compared with the LSP50 panel. This indicates that an increase in the RHB content results in panels that are less resistant and less tough.

#### 3.3.3. Failure Mode of Lightweight Sandwich Panels

According to [49], four primary collapse modes have been identified for sandwich beams under three-point and four-point bending: (a) Mode 1: face yielding or face microbuckling, (b) Mode 2: wrinkling of the compressive face sheet, (c) Mode 3: core shear, and (d) Mode 4: indentation beneath the loading rollers. In this study, the failure mode of the sandwich panel was influenced by RHBC core. In addition to the properties of the materials comprising the sandwich panel, its failure mode when subjected to bending is also influenced by geometric aspects, such as core thickness [49], and by testing conditions, including the type of flexural test [50].

**Figure 16:**
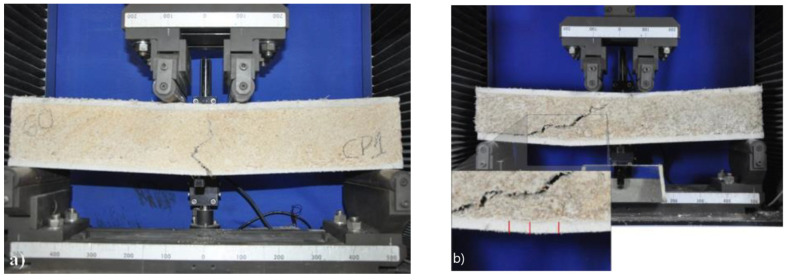
(**a**) Typical failure mode of lightweight sandwich panels LSP50 and LSP6, and (**b**) typical failure mode of lightweight sandwich panel LSP70.

For samples submitted to long beam flexure, the failure mode of LSP50 and LSP60 was initially characterized by face yielding, primarily in the form of crack opening, which then propagated through the core of the lightweight sandwich panel, as shown in Figure 16a. A similar failure mode was observed by Priyanga [19] when analyzing textile-reinforced concrete sandwich panels under bending; after the first crack in the face, crack propagation through the core was observed. In the lightweight panels using ultra-high-performance fiber-reinforced concrete, as evaluated by Ghazy [51], the primary failure mode for the sandwich specimens began with a vertical crack forming at the midspan of the core layer, which grew as the load increased.

For LSP70, only one crack formed, with no new cracks observed. The F_u_ value for LSP70, presented in Table 3, indicates a lower stress level on the face of the sandwich lightweight panel compared with LSP50 and LSP60. Upon evaluating the lateral cracking pattern of this panel, it was noted that, due to the lower compressive strength of the core, a shear crack developed between the supports and the load application points, as shown in Figure 16b. Consequently, Mode 3 failure, characterized by shear failure of the core, was predominant.

Colombo et al. [52] evaluated sandwich panels with textile-reinforced concrete faces and an EPS core, with spans of 0.5 and 1 m. For the large panels, it was observed that, after the faces cracked, the failure mode was characterized by the formation of a shear crack starting from the support, similar to the behavior observed in the LSP70 panel.

An important aspect observed in the failure of sandwich panels was the absence of delamination between the core and the face, indicating good face–core adhesion. Pull-out tests conducted by Frazão [23] in specimens extracted from sandwich panels with cement-based face and core showed that failure occurred due to cohesion, and not due to the interface–core debonding.

With a modification of testing configuration, a change in the failure mode of the panels was observed, as shown in Figure 15, which occurred by core shear instead of the propagation of a flexural crack in the center of the core (Figure 16). The results confirm the hypothesis presented by [49] that, for short spans (with same position of loading), core failure occurs first and then failure in the face.

In the LSP50 and LSP60 panels under shear, failure occurs in Mode 3, characterized by core shear. As the panel displacement increased, a widening of the inclined crack between the support and the load application point was observed, which is characteristic of shear failure in beams. For larger displacements, around 3 to 4 mm, cracks began to appear on the tensioned faces, both at the support point and between the supports. This failure mode is different from what was previously observed (Table 3). With the reduction in the span and the distance between the points of load application and supports, there was a reduction in the acting bending moment and the tensile stresses on the lower face. Thus, the shear stresses that arise in the central region of the cross-section govern the failure mode of the lightweight sandwich panel.

For the LSP70 panel, the lower core strength leads to the same failure mode observed in the bending test, characterized by core compression crushing. As shown in Figure 15c, even at large displacements, no cracks were observed in either the core or the faces. According to [45], the failure mode of a sandwich panel can vary from core shear to core crushing in tests with the same free span length, depending on the density of the core material.

## 4. Conclusions

In this study, a new type of lightweight sandwich panel was introduced, consisting of foamed cement-based materials reinforced with short sisal fibers for the faces, and a core made of RHBC.

The use of a foaming agent allows the production of lightweight cementitious composites with reduced density and compressive strength; however, an increase in flexural strength was observed. This improvement is attributed to the fibers’ action after the first crack appears in the matrix, as they inhibit crack propagation and enhance the load-bearing capacity. Based on the efficiency factor evaluation, the composite with 20% foaming agent was found to be the most suitable for use on the face of the sandwich panel.

To produce a sustainable and lightweight core, RHB was used in the production of concrete at proportions of 50%, 60%, and 70%, resulting in a material with densities below 800 kg/m^3^ and compressive strength ranging from 0.35 to 1.92 MPa.

Three types of sandwich panels were produced with different core types, containing 50%, 60%, and 70% RHB. All of them maintained a density below 1000 kg/m^3^, classifying them as lightweight panels. The mechanical behavior of the lightweight panels was evaluated through flexural tests with spans of 200 mm and 500 mm, which allowed for the assessment of the face’s resistance to normal stress and the core’s shear resistance.

The experimental mechanical results demonstrate that the lightweight panels exhibited a deflection–hardening behavior, with a linear section until the appearance of the first crack, followed by an increase in load-bearing capacity. This increase in flexural strength, which reached approximately 3 MPa, was made possible by the presence of fibers in the faces, which inhibited crack propagation and allowed for stress redistribution. On the other hand, the maximum load-bearing capacity was also directly affected by the strength of the RHBC core. The increase in RHB content resulted in a reduction in both the ultimate stress on the face (Fu), the core shear ultimate stress (Fs), and the toughness of the panels. The values of Fu and Fs decreased by about 87% when the RHB content in the core increased from 50% to 70%. A reduction of up to 83% was also observed in the toughness of the lightweight sandwich panels with changes in the core concrete type.

Due to the lower mechanical strength and stiffness, the concrete core with a higher RHB content was unable to transfer stresses between the two faces of the sandwich panel, preventing it from functioning as a fully composite structure. This affected both the load-bearing capacity of the sandwich panel and its failure mode. Under flexion, the sandwich panels with 50% and 60% RHB exhibited a failure mode characterized by face yielding, while in the panel with 70% bio-aggregate, the failure occurred due to the propagation of a shear crack between the support and the load application point. The shear failure of the sandwich panels was also influenced by the RHB content, with a shift from a shear core failure mode to a crushing core failure mode when a higher RHA content was used.

The incorporation of biomaterials in both the faces and the core of lightweight sandwich panels promotes sustainability in the construction sector by utilizing renewable, biodegradable materials with a low energy production cost. This new construction element shows good potential for application in roofing and insulation walls. However, the results also show that the RHB content plays a key role in determining the panel’s intended application, as the reduction in density leads to a decrease in mechanical strength and alters the failure mode of the panels.

## Figures and Tables

**Table 1:** RHBC mix design (kg/m^3^).

Mix	C	MK	FA	RHB	W	WC	VMA	CaCl_2_
RHB50	320.5	160.2	53.4	150.0	438.1	171.0	0.53	16.0
RHB60	256.4	128.2	42.7	180.0	418.9	205.2	0.42	12.8
RHB70	192.3	96.1	32.0	210.0	400.0	239.4	0.32	9.61

C = cement; MK = metakaolin; FA = fly ash; RHB = rice husk bio-aggregate; W = water; WC = water for compensation; VMA = viscosity agent.

**Table 2:** Mechanical properties of composites.

Mix	Foam Volume (%)	Compressive Strength (MPa)	Elastic Modulus (GPa)	Flexural Strength (MPa)
SFRC0	0	33.53 (3.4)	15.76 (1.7)	4.16 (3.0)
SFRC15	15	27.68 (2.8)	12.77 (9.0)	5.12 (2.7)
SFRC20	20	20.81 (5.6)	11.50 (8.1)	5.54 (4.0)
SFRC30	30	15.37 (8.9)	7.70 (4.1)	3.60 (4.6)

**Table 3:** Flexural properties of lightweight sandwich panels under flexion.

Panel	Bio-Aggregate Content (%)	F_fcr_ (kN)	F_max_ (kN)	δ_max_ (mm)	F_u_ (MPa)	Toughness (kN·mm)	Failure Mode
LSP50	50	1.08 (4.6)	2.21 (5.3)	0.75 (11.03)	3.07 (5.3)	5.45 (14.0)	Face yield
LSP60	60	0.90 (9.4)	1.62 (1.7)	0.97 (7.13)	2.25 (1.7)	4.80 (28.4)	Face yield
LSP70	70	0.28 (10.0)	0.31 (6.1)	0.41 (13.21)	0.43 (6.1)	1.29 (15.3)	Shear core

**Table 4:** Flexural properties of lightweight sandwich panels under shear test.

Panel	Bio Aggregate Content (%)	F_max_ (kN)	δ_max_ (mm)	F_s_ (MPa)	Toughness (kN·mm)	Failure Mode
LSP50	50	5.40 (6.3)	0.27 (7.9)	0.37 (6.3)	17.81 (10.2)	Shear core
LSP60	60	2.72 (11.8)	0.78 (10.5)	0.18 (11.8)	10.67 (11.9)	Shear core
LSP70	70	0.67 (9.0)	0.80 (6.0)	0.05 (9.0)	2.95 (11.9)	Crushing core

## Data Availability

The original contributions presented in this study are included in the article. Further inquiries can be directed to the corresponding author.
